# Temperature-Responsive Microrobot for High-Temperature Sensing in Constrained Environments

**DOI:** 10.34133/research.0760

**Published:** 2025-07-04

**Authors:** Shaobo Ding, Junmin Liu, Jiaxu Dong, Rencheng Zhuang, Enbo Shi, Shutong Wang, Yuhang Xiao, Dekai Zhou, Longqiu Li, Xiaocong Chang

**Affiliations:** ^1^ Key Laboratory of Microsystems and Microstructures Manufacturing (Harbin Institute of Technology), Ministry of Education, Harbin 150001, China.; ^2^ Zhengzhou Research Institute of Harbin Institute of Technology, Zhengzhou 450000, China.; ^3^ Chongqing Research Institute of Harbin Institute of Technology, Chongqing 400722, China.

## Abstract

Temperature measurement in confined environments has long been a substantial challenge. Due to poor accessibility to constrained space, and low visibility, conventional thermometry and existing nanoscale thermometers struggle to achieve efficient and accurate temperature detection. As an emerging technology, microrobots offer great potential for temperature sensing in such challenging conditions. Here, we propose a temperature-responsive microrobot (TRM) that integrates artificial neural networks into microscale thermal sensing, enabling quantitative temperature measurement in complex and constrained environments. The TRM undergoes irreversible color changes in a high-temperature range of 160 to 240 °C. It features a Janus structure composed of a Cu(NH_3_)_4_SO_4_-based thermochromic material and a nickel-coated magnetic actuation layer, allowing reliable operation in nontransparent and geometrically confined environments such as porous geological structures and constrained microspaces. The thermochromic mechanism and motion dynamics of the TRM under elevated temperatures were systematically investigated. The microrobot exhibits distinct chromatic responses at different temperatures. Based on the correlation between chromaticity and temperature, a multilayer perceptron neural network was developed. By inputting the observed color features into the trained model, the surrounding temperature can be quantitatively determined. Experimental results in a simulated porous microchannel model confirmed the feasibility and effectiveness of the TRM for localized high-temperature detection. This work provides a new solution for temperature sensing in restricted environments and lays a solid foundation for the application of microrobots in industrial high-temperature monitoring, highlighting their potential for real-world deployment in complex conditions.

## Introduction

Temperature sensing plays a pivotal role in controlling and monitoring industrial, chemical, and biomedical systems [1]. There are some complex internal space structures such as porous and zigzag in microscale or constrained environments. Traditional temperature measurement techniques such as thermocouples [2,3], infrared sensors [4–6], and fiber optics [7–9] face larger challenges in such specific environments. This is not only due to their size but also because of limitations in mechanical flexibility, spatial adaptability, or contact requirements restrictions. To overcome these challenges, researchers have explored various nanoscale thermometers, including quantum dots [10–12], lanthanide-doped nanoparticles [13,14], and nitrogen-vacancy (NV) centers in diamond [15–17]. These systems exhibit high spatial resolution and, in some cases, extended temperature capabilities up to 500 °C. Despite their precision, they typically require continuous optical excitation and real-time fluorescence detection, rendering them unsuitable for use in nontransparent or visually inaccessible environments such as geological porous structure or embedded structural systems. Micro-nanorobots, with their small size and autonomous mobility, have the potential to act as mobile sensors in complex media, making them particularly suited for performing sensing tasks at the micro- and nanoscale. The use of micro-nanorobots for temperature sensing in narrow, confined spaces offers considerable application potential.

Micro-nanorobots can be driven by various external fields, including chemical [18–20], electric fields [21–23], ultrasound [24–27], magnetic fields [28–31], and light fields [32–36]. These micro-nanorobots have been widely explored in the fields of biomedical and environmental monitoring due to their advantages in active detection and precise target positioning [37–41]. Based on the sensing principles, current research can be roughly classified into 3 categories: dynamic feature sensing, static feature sensing, and auxiliary sensing [42–46]. Dynamic feature sensing refers to the perception of environmental information by micro-nanorobots through changes in speed [47–49], deformation [50–52], and other dynamic responses during motion or under force. For example, micro-nanorobots labeled with silver nanoparticles capture target nucleic acids, releasing silver ions into hydrogen peroxide solution, thus increasing the robot’s speed to detect DNA or bacterial RNA [47]. Hydrogen peroxide catalase decomposes H₂O₂ to produce bubbles that propel micro-nanorobots; in the presence of heavy metals or pesticides, the bubble production rate decreases, allowing water quality monitoring [49]. Magnetic-field-induced ferromagnetic fluid droplets elongate into ellipsoids under the influence of a magnetic field, and their strain correlates with the mechanical properties of surrounding materials, demonstrating the feasibility of using ferromagnetic fluid droplets as micro-rheometers and micro-tensiometers [50]. Additionally, the reversible reconstruction ability of vortex-like nanoparticle assemblies enables the sensing of local fluid viscosity and ion strength [49], making micro-nanorobots capable of precisely measuring fluid properties such as the viscosity and ion strength of whole pig blood [53]. The movement of micro-nanorobots greatly enhances the response and sensitivity of the detection. Auxiliary sensing refers to the enhancement of the performance of other sensing technologies by micro-nanorobots, such as improving molecular diffusion, enhancing signals, or serving as carriers to increase sensitivity [54,55]. For example, micro-nanorobots manufactured via nanoimprint lithography substantially improve surface-enhanced Raman scattering signals by optimizing surface plasmonic nano-structures, thus enhancing the detection of low-concentration analytes. Xiong et al. [56] developed dynamic magnetic nanoparticles that actively mix and eliminate the slow diffusion rate of molecules from solution to probe surfaces, enhancing the sensitivity of DNA and protein analysis and accelerating the detection process. Static feature sensing refers to the detection of environmental static physical or chemical characteristics, such as fluorescence [57–61], electro-luminescence [62], and visible color changes [63]. For example, micro-nanorobots encapsulated in temperature-responsive or pH-responsive hydrogel shells undergo noticeable fluorescence color changes under optical microscopy when exposed to different temperatures or pH values, thus enabling temperature or pH sensing [59–63]. It is also possible to carry thermoluminescent materials on micro-robots to achieve temperature sensing [64]. Glucose in a solution can be oxidized by glucose dehydrogenase, converting NAD^+^ to NADH and promoting electrochemical luminescence (ECL) of Ru(bpy)_3_^2+^. The intensity of ECL changes with the glucose concentration, allowing glucose concentration monitoring. In cortisol immunoassays, cortisol-horseradish peroxidase conjugated with anti-cortisol functionalized micro-nanorobots results in a visible deep blue tetramethylbenzidine color, enabling visual detection of H₂O₂. Although microrobot temperature sensing has been explored using fluorescence and reversible thermochromic materials, such approaches often require sophisticated detection setups and remain constrained to low-temperature regimes.

**Fig. 1.**
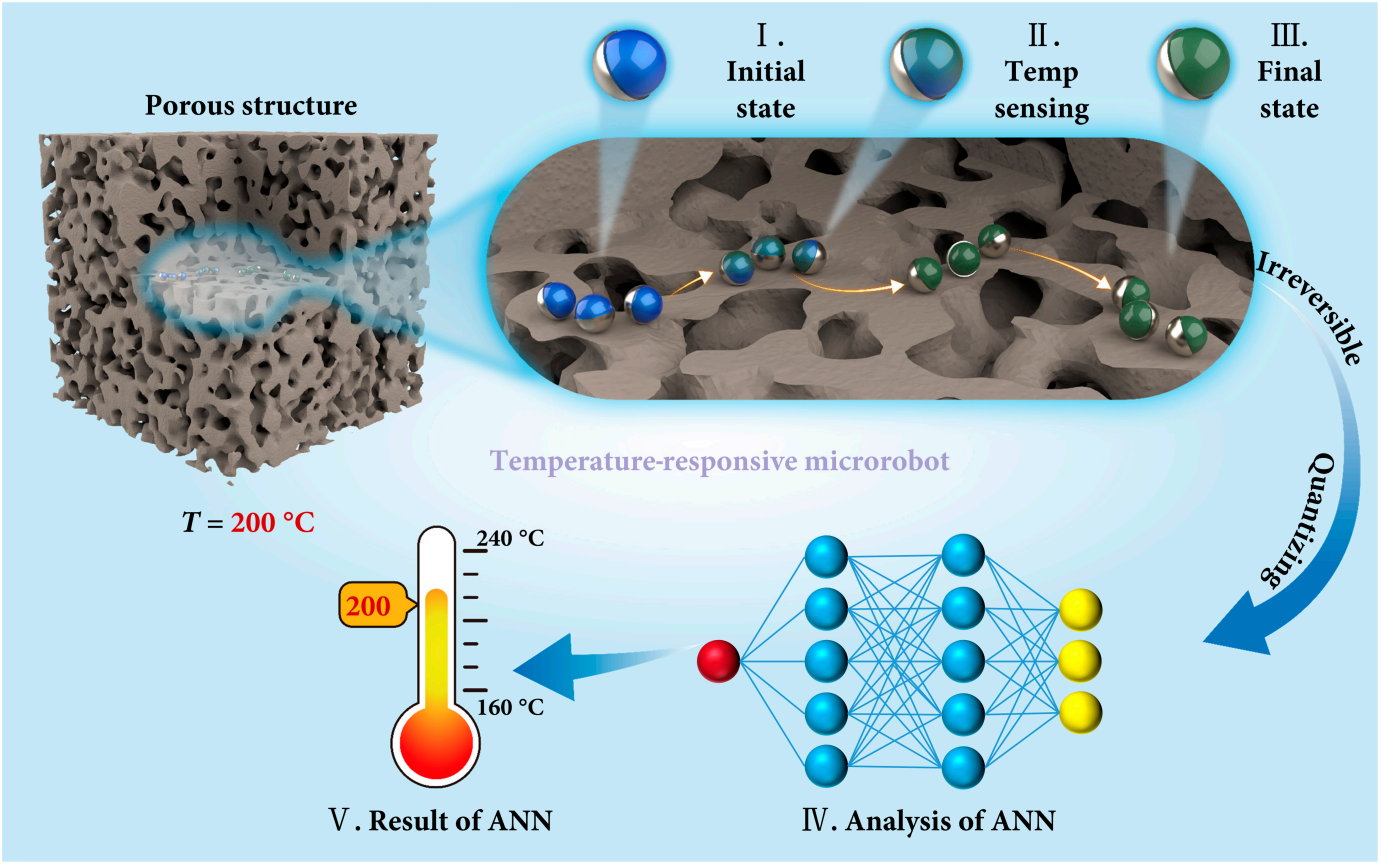
Schematic illustration of TRM sensing the internal temperature of a micro-porous core.

In this study, we propose a temperature-responsive microrobot (TRM) that integrates artificial neural networks into microscale temperature sensing, enabling both operation and temperature detection in high-temperature environments. By systematically investigating its temperature-sensing mechanism, fabrication process, and magnetic actuation strategy, the TRM achieves reliable temperature detection and controllable motion under extreme thermal conditions. The microrobot features a Janus structure, with one-half composed of magnetic material and the other half composed of temperature-sensitive material. This configuration exhibits distinct chromatic responses at different temperatures. Moreover, the direction and speed of its motion can be flexibly regulated by adjusting the orientation, strength, and frequency of the external magnetic field. As illustrated in Fig. 1, the TRM can be injected into a high-temperature region with a micro-scale spatial structure of tiny voids. Initially, the TRM exhibits its ambient temperature color. After moving through the targeted high-temperature zone, the microrobot can detect the temperature and change its color to an irreversible color according to the highest temperature. This color change recorded the temperature information of the high-temperature region. The TRM then exits the micro-scale spatial structure and is collected. A multilayer perceptron (MLP) neural network model analyzes the recorded color changes to determine the specific temperature of the high-temperature zone, thereby completing the entire sensing task. This process eliminates the need for real-time power supply or in-situ optical monitoring. Compared with both conventional and nanoscale methods, our approach uniquely combines magnetic actuation, passive operation, irreversible high-temperature sensing, and neural network analysis, making it particularly suitable for temperature monitoring in structurally complex or geometrically confined environments where traditional sensors are difficult to deploy. A detailed comparison is summarized in Table S1.

## Results and Discussion

### Sensing mechanism, fabrication, and characterization of TRMs

**Fig. 2.**
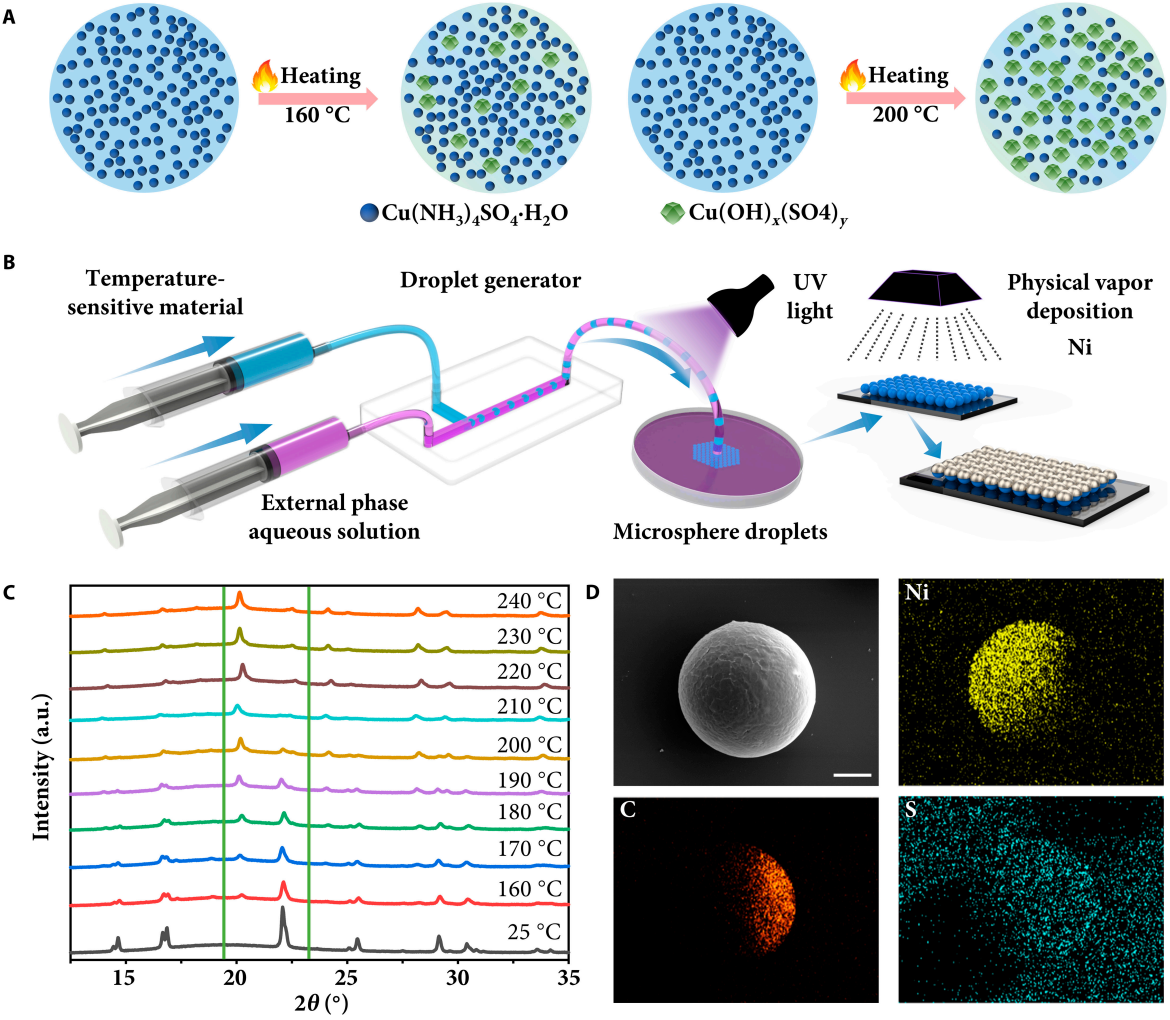
Mechanism and fabrication of TRM. (A) Schematic illustration of the TRM temperature-sensing mechanism. (B) Schematic of the TRM fabrication process. (C) XRD analysis of the base microspheres. (D) SEM images and energy-dispersive x-ray spectroscopy (EDS) analysis of TRMs. Scale bar represents 25 μm.

The temperature sensing mechanism of the TRM relies on the color change of its temperature-sensitive material before and after thermal exposure. This colorimetric transformation, induced by thermal decomposition of tetraamminecopper(II) sulfate and subsequent polymer oxidation, enables visual estimation and quantitative analysis of the peak temperature during sensing. Initially, Cu(NH_3_)_4_SO_4_ exhibits a bright blue color, while the photocurable resin remains colorless. As shown in Fig. 2A, upon heating, the blue Cu(NH_3_)_4_SO_4_ undergoes a transformation, gradually converting into greenish Cu(OH)*_x_*( SO_4_)*_y_*. This transition begins at approximately 150 °C, but with a minimal conversion rate, resulting in only a slight color change. From 160 °C onward, the color change becomes more pronounced as the conversion rate increases, progressively shifting toward the characteristic hue of basic copper sulfate. By 200 °C, the transformation is nearly complete. At temperatures above 200 °C, the temperature response of the TRM is primarily attributed to oxidative reactions involving weak sites within the polymer chains of the resin. These reactions with oxygen generate chromophoric structures such as conjugated double bonds and carbonyl groups, which exhibit strong absorption in the 400- to 500-nm wavelength range, leading to a visually perceptible yellowing effect.

Notably, the thermochromic transition did not initiate appreciably within the first 3 min of exposure at 160 °C. This observation is attributed to the inherent activation energy barrier associated with the thermal decomposition of tetraamminecopper(II) sulfate and the subsequent oxidation of the polymer matrix. During this initial phase, although the temperature is sufficient to initiate molecular vibration, the conversion of Cu(NH_3_)_4_^2+^ complexes to basic copper sulfate Cu(OH)*_x_*(SO_4_)*_y_* remains thermodynamically unfavorable. These changes are slow to initiate. The sensing duration was fixed at 4 min to ensure the onset of a distinct color change. This also helps minimize the sensing time and enhance the efficiency of temperature detection.

The fabrication of TRMs primarily employs droplet-based microfluidic technology, which generates dispersed microdroplets by manipulating immiscible continuous and dispersed phases [65]. As illustrated in Fig. 2B, spherical droplets with temperature-sensing functionality are first prepared using a droplet generator. The specific experimental setup is shown in Fig. S1. The generator is designed with a T-shaped channel structure to facilitate droplet generation. Due to the high viscosity of the dispersed phase solution, the channel dimensions are optimized to prevent clogging. The dispersed phase channel has a width of 500 μm, while the continuous phase channel width is 800 μm, with both channels having a depth of 500 μm. At the outlet of the continuous phase, the droplets are solidified under ultraviolet irradiation at a wavelength of 395 nm, resulting in the formation of TRM base microspheres. To achieve rapid and large-scale fabrication of microspheres, the dispersed phase flow rate is set to 100 μm/min, while the continuous phase flow rate is maintained at 10 ml/min. The ultraviolet light power is 20 × 3 W, and the exposure time is 30 s. To eliminate potential interference from the continuous phase solution on the sensing performance of TRMs, the obtained base microspheres underwent a separation process to remove residual continuous phase solution. This is achieved by washing them with anhydrous ethanol 2 to 3 times until the continuous phase was completely removed. Subsequently, the purified microspheres are evenly spread onto a glass slide. A physical vapor deposition (PVD) method is used to coat the surface of the microspheres with a 300-nm-thick nickel layer. This step ensures that the TRMs have the required structural and functional properties for effective temperature sensing and motion. The microscopic morphology of the microspheres is shown in Fig. S2.

As shown in Fig. 2C, the x-ray diffraction (XRD) analysis of the base microspheres at different temperatures demonstrates their temperature-dependent structural evolution. Within the scanning angle range of 15° to 25°, the original diffraction peaks gradually weaken. Meanwhile, new diffraction peaks emerge between 160 and 240 °C, corresponding to the thermochromic transition from blue to yellow. For other characterizations of base microspheres (Figs. S3 and S4). This irreversible phase transition highlights the material’s sensitivity to thermal stimuli. The fabricated microrobots were characterized using scanning electron microscopy (SEM) and energy-dispersive x-ray spectroscopy (EDS), as shown in Fig. 2D. The SEM images provide a clear depiction of the surface morphology of the microspheres. The EDS analysis reveals distinct elemental signals, with a strong Ni signal confirming the successful and uniform deposition of a nickel layer on the microsphere surface via PVD. Additionally, the presence of S indicates the effective integration of Cu(NH_3_)_4_SO_4_ into the microsphere structure, which is essential for the microrobot’s temperature-sensing functionality. The detection of C further validates the incorporation of the high-temperature-resistant photocurable resin, which plays a crucial role in enhancing the thermal stability and functionality of the microrobot. These results collectively demonstrate the successful fabrication and functional integration of the microrobot, ensuring its suitability for high-temperature sensing applications. In addition, we can investigate a wide range of thermochromic material types and the proportions of each type to achieve broader temperature ranges. We give a simplified example in Fig. S5.

### Motion of the TRMs

**Fig. 3.**
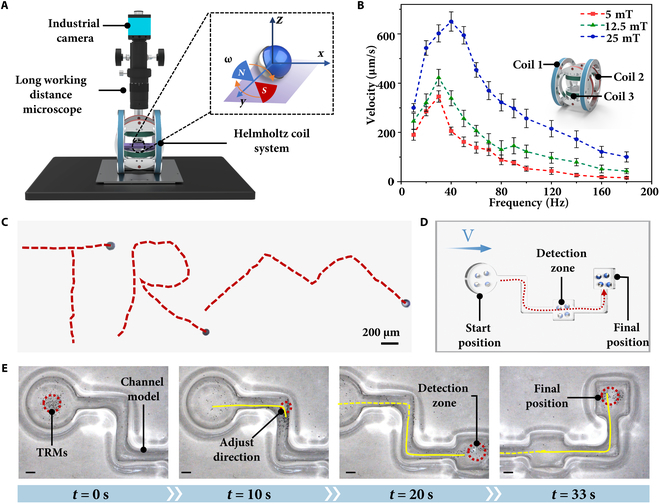
Motion behavior of TRMs. (A) Schematic illustration of the TRM motion control setup. (B) Speed variation of the TRM under different magnetic field strengths and frequencies. (C) Trajectory of the TRM (Movie S1). (D) Schematic diagram of the TRM simulation execution task. (E) Trajectory and temporal sequence of the TRMs during motion (Movie S2). All scale bars represent 800 μm.

Efficient motion control is a critical aspect of ensuring that TRMs can navigate complex environments to perform localized temperature sensing. This section examines the motion behavior of TRMs under magnetic field control. It highlights their trajectory and responsiveness in dynamic scenarios. As shown in Fig. 3A, the experimental setup for evaluating the locomotion capability of the TRM consists of an industrial camera, a long working distance microscope, and a custom-built 3-axis Helmholtz coil magnetic control system (Fig. S6). This Helmholtz coil system comprises 3 coils that generate a uniform rotating magnetic field, enabling precise control over the TRM’s motion.

To further investigate the locomotion characteristics of the TRM, we first examined the relationship between its velocity and the applied magnetic field frequency and strength. Figure 3B illustrates the effects of magnetic field frequency and strength on the motion speed of TRMs under identical medium conditions (air). The study revealed that the motion speed of TRMs increases markedly with higher magnetic field strength. Specifically, as the field strength increases from 5 to 25 mT, the peak velocity of TRMs rises from 346 to 650 μm/s, demonstrating a clear linear enhancement trend. In addition to field strength, the frequency of the magnetic field also plays a critical role in determining motion speed. When the frequency increases from 10 to 180 Hz, a peak frequency ($f_{p}$) corresponding to the maximum speed is observed. For $f<f_{p}$, the motion speed increases with frequency; however, for $f>f_{p}$, the speed decreases as the frequency further increases. The occurrence of the peak frequency (also referred to as the step-out frequency) is due to the frequency lag inherent in microrobot systems. When the magnetic field strength and the viscosity of the surrounding medium remain constant, the microrobots rotate at a limited angular velocity. As the frequency of the applied magnetic field exceeds this limit, the microrobots begin to fall out of synchrony, resulting in increasing phase lag and a gradual decrease in rolling speed until motion stops entirely. The step-out frequency increases with magnetic field strength, as a stronger magnetic field generates a higher magnetic torque, thereby raising the upper limit of angular velocity and enabling the microrobots to follow higher-frequency fields. As shown in the figure, the step-out frequency increases from 30 Hz at 5 mT to 40 Hz at 25 mT. These findings underscore the advantages of the TRMs in achieving tunable motion performance under varying magnetic field conditions, providing valuable insights for optimizing magnetic control designs and expanding their practical applications.

Subsequently, we validated the precise locomotion capability of the TRM. By controlling its trajectory in a fluid medium, we guided the TRM to trace the letters “TRM”. It demonstrates the ability to achieve precise and controllable motion under an applied magnetic field. Furthermore, under optimized magnetic field frequency and strength, we designed a glass channel model to simulate the task execution process of the TRM, as illustrated in Fig. 3D. Figure 3E illustrates the temporal sequence and trajectory of TRMs during movement within the channel, clearly showcasing their dynamic motion characteristics. At *t* = 0 s, TRMs are located at the starting position; at *t* = 10 s, they make their first directional adjustment, demonstrating their ability to respond to the external rotating magnetic field and execute precise turns; at *t* = 20 s, TRMs reach the target detection area and successfully localize; and at *t* = 33 s, they arrive at the endpoint of the channel following the designated path. Throughout the motion, TRMs exhibit high precision and directional control, with smooth rolling trajectories free of appreciable deviations or interruptions. Further analysis indicates that the TRM trajectories are influenced by the synergy between the magnetic field strength and channel design, resulting in stable, smooth movement. The consistent performance across time points validates the reliability and robustness of the system design.

### Training of the TRM sensing model

Building on the temperature-sensing mechanism of TRMs, this section describes the development of a neural network-based sensing model. The integration of experimental data and advanced machine learning techniques provides a framework for accurate and efficient temperature prediction. In the experiment, the prepared TRMs were placed on a glass slide and heated under controlled laboratory conditions. The heating temperature ranged from 160 to 240 °C, with 10 °C increments, and each temperature was maintained for 4 min. After heating, the TRMs were observed under a microscope, and high-resolution color images corresponding to each temperature were captured using an ultra-depth digital microscope. The lighting setup involved using the default illumination settings and halo-removing mode of the microscope. To ensure data reliability and representativeness, multiple images were recorded at each temperature, collecting color data from 100 individual TRMs. The experimental results revealed a pronounced color transition in the TRMs as the temperature increased, which closely aligned with the color-changing mechanism of the temperature-sensitive material. At room temperature, the TRMs appeared bright blue. When heated to 160 °C, the color gradually shifted toward green and deepened with increasing temperature. Beyond 200 °C, the TRM color started transitioning from green to yellow, eventually turning deep yellow at 240 °C. These observations confirm the potential of TRMs for precise temperature sensing through visually distinguishable color changes, providing a solid experimental foundation for their application in high-temperature environments.

**Fig. 4.**
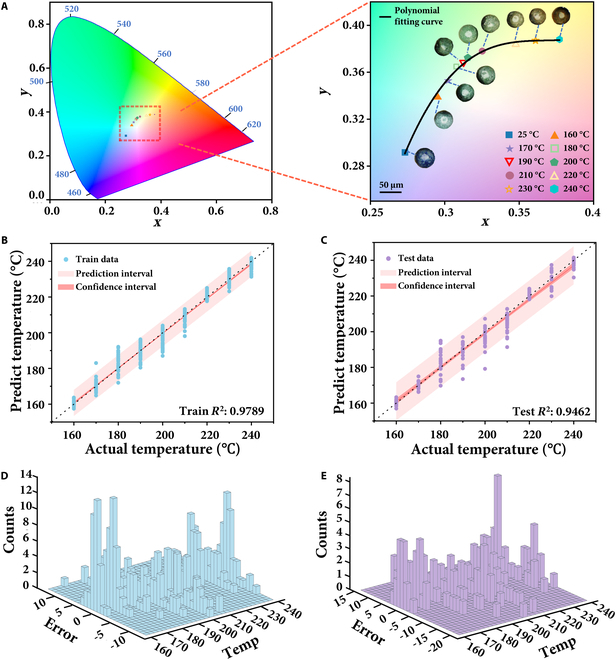
Temperature sensing performance of TRMs. (A) Calibration of TRM colorimetric response before and after temperature sensing in the CIE 1931 color space. (B) Performance of the neural network on the training dataset. (C) Performance of the neural network on the test dataset. (D) 3D residual plot of the training dataset. (E) 3D residual plot of the test dataset.

To quantitatively evaluate the color characteristics of TRMs, a systematic color analysis was performed on microscopic images obtained at different temperatures. Given the random orientations of TRMs in the experiments, only the non-nickel-coated regions were analyzed to avoid interference from the nickel layer. First, Canny edge detection was applied to segment the TRMs from the background. Subsequently, the color information of the non-nickel-coated regions was extracted using the HSV color space thresholding method, and the corresponding RGB values of TRMs at different temperatures were obtained (Fig. S7). In addition to standard color feature extraction, we also considered potential occlusion scenarios during TRM operation. In practical deployments where TRMs may be partially covered by debris or structures, the same algorithm can be used to extract temperature features from visible portions. To visually illustrate the color transition trend of TRMs, the average RGB values at each temperature were converted into chromaticity coordinates and mapped onto the CIE 1931 color space, as shown in Fig. 4A. Different markers, varying in shape and color, represent different temperatures, while the curve depicts the corresponding trend obtained through polynomial fitting. The experimental results indicate that the *x*-coordinate of the chromaticity values ranges from 0.25 to 0.4. As the temperature increases, both the *x*- and *y*-coordinates of the chromaticity values exhibit an upward trend. Correspondingly, the TRM color transitions from bright blue to green and ultimately to yellow. This pronounced color change highlights the high sensitivity of TRMs to temperature variations, demonstrating their potential for precise and visually distinguishable temperature sensing in dynamic environments.

In order to establish a predictive relationship between color features and temperature, we developed an MLP neural network regression model that utilizes the extracted color characteristics of TRMs and their temperature-dependent transitions. Specifically, the model takes the average RGB values of TRM images as input features and the corresponding temperature as the output variable. Both input and output data were standardized to ensure consistency and stability during training. The dataset was divided into 2 subsets, with 70% used for training and 30% reserved for independent testing to evaluate the model’s generalization capability. The architecture of the neural network is as follows: the input layer consists of 3 nodes (corresponding to the RGB channels), followed by 3 hidden layers with 256, 128, and 64 neurons, respectively, and a single-node output layer (representing the predicted temperature). The ReLU activation function was used in the hidden layers (as shown in Eq. 1), while the mean squared error loss function was employed (Eq. 2). The Adam optimizer was selected for parameter optimization. This optimized neural network framework effectively maps color features to corresponding temperature values, providing a robust and efficient model for temperature sensing based on TRM colorimetric response.

$$ReLU\left(x\right)=max\left(0,x\right)=\left\{\begin{array}{c} x,\text{if}\;x>0\\ 0,\text{if}\;x\leq 0 \end{array}\right.$$ (1)

$$\mathrm{Los}s_{\mathrm{MSE}}=\frac{1}{N}\sum_{i=1}^{N}{\left(y_{i}-{\hat{y}}_{i}\right)}^{2}$$ (2)

where N is the total number of samples, $y_{i}$ is the actual value of the ith sample, and ${\hat{y}}_{i}$ is the predicted value for the sample.

The performance of the trained model was evaluated using both the training and testing datasets. As illustrated in Fig. 4B, the model’s predictions for the training dataset closely align with the ideal values, achieving a coefficient of determination ($R^{2}$) of 0.9789. Similarly, Fig. 4C shows the prediction results for the testing dataset, where the $R^{2}$ value remains at 0.9462. In both Fig. 4B and C, the pink region corresponds to the 95% prediction interval, and the deep pink region represents the 95% confidence interval. Figure 4D and E illustrate the residual distribution at different temperatures after model training. It can be observed that the errors for both the training and test sets are concentrated within the range of −5 to 5, and the residuals at each temperature point approximately follow a normal distribution. This indicates that the model exhibits good predictive accuracy across different temperature ranges, with uniformly distributed errors and no appreciable systematic bias. Furthermore, the near-normal distribution of residuals suggests that the model effectively captures the mapping between temperature and color features, without showing signs of overfitting or underfitting. These findings validate the reliability and applicability of the MLP neural network in temperature prediction based on the color response of TRMs, laying the foundation for their application in intelligent temperature sensing.

With the trained neural network, temperature sensing becomes markedly more efficient. The process involves capturing an image of the microrobot after it has been heated to a specific temperature, inputting the image into the neural network model, and obtaining the corresponding temperature as the output. This streamlined approach demonstrates the practicality and precision of the neural network model for temperature prediction in microscale environments.

### Experimental model validation of TRM sensing

**Fig. 5.**
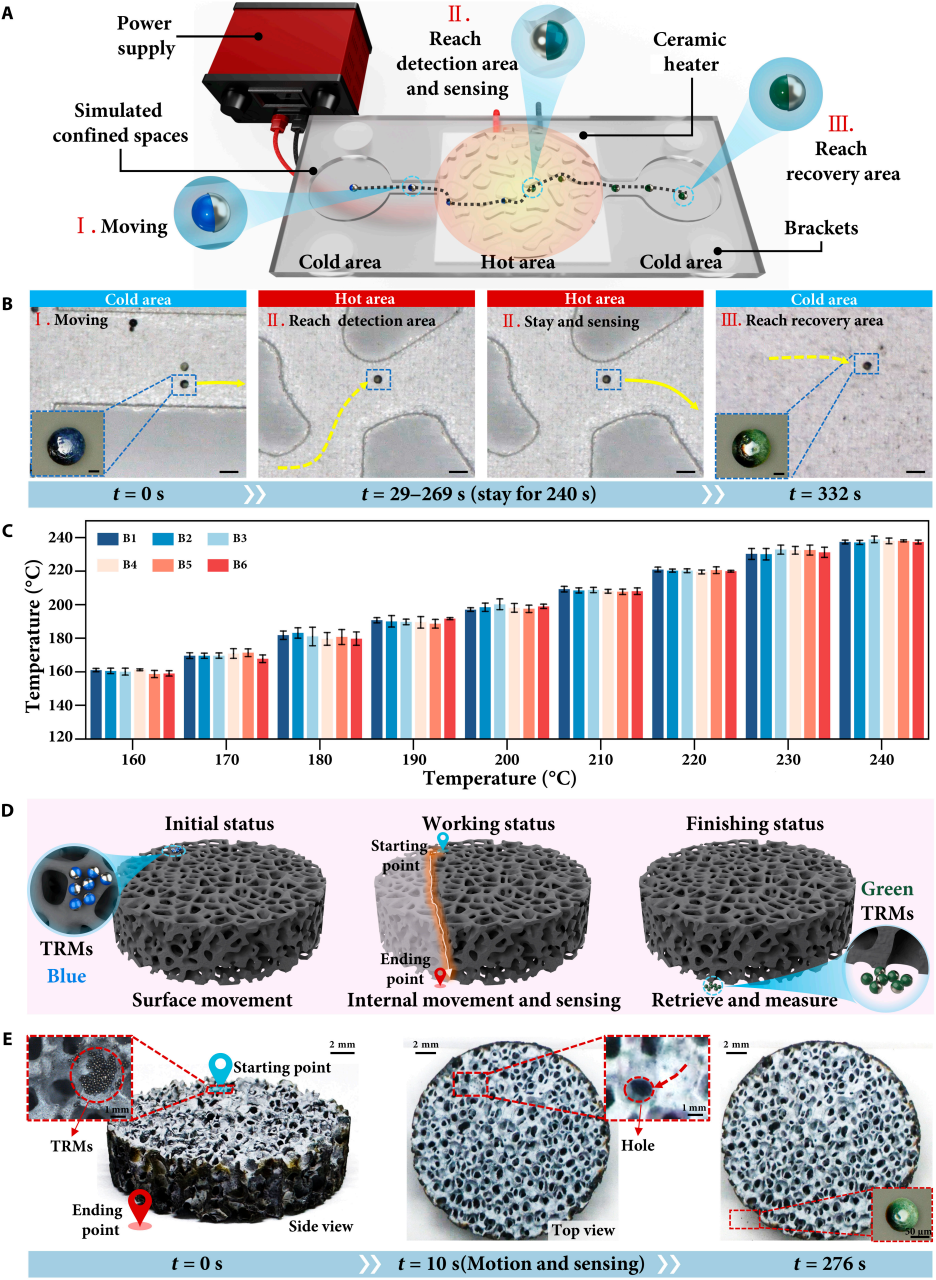
TRM simulated core temperature measurement. (A) Schematic diagram of TRM measuring internal temperature within a simulated a micro-porous core. (B) Temporal sequence and trajectory of TRM during the internal temperature measurement in the simulated micro-porous core. Scale bars of normal figure represent 200 μm; scale bars of enlarged figure represent 25 μm. (C) Results of TRM stability validation under identical conditions. (D) Schematic of temperature sensing of TMRs in porous structures. (E) Temporal sequence and trajectory of TRM during the bottom temperature measurement in the porous silicon carbide (Movie S6).

A simplified experimental setup was designed to simulate high-temperature environments in confined spaces, to evaluate the practical applicability of TRMs. This section highlights the temperature sensing capability of TRMs, emphasizing their reliability and potential industrial applications. The experimental setup was meticulously configured to replicate high-heat conditions in restricted spaces, enabling controlled validation of TRM performance. For the convenience of observation, the experimental setup was designed to simulate a cross-sectional perspective of a confined interior environment. As shown in Fig. 5A, the system consists of a 12-V power supply, a 2-dimensional confined-space simulator fabricated via laser processing of glass, a ceramic heating element, a support structure, and the previously described 3-dimensional (3D) Helmholtz coil magnetic control system. During the experiment, the TRM was initially placed in a lower-temperature region and guided by a controlled magnetic field to the target sensing area. After temperature detection, the TRM was directed to a designated recovery zone, where its color change was captured using an ultra-depth digital microscope for further analysis. Before the experiment, the ceramic heater elevated the target area temperature to 200 °C to ensure reliable and repeatable performance assessment of the TRM under simulated environmental conditions. The temperature control is achieved by adjusting the supply voltage of the ceramic heating element, where different voltages correspond to different temperatures. Temperature measurement is conducted using an infrared thermometer (Fig. S8), and experiments are performed only after the temperature has stabilized.

The sensing process was monitored and recorded, with key snapshots displayed in Fig. 5B. At *t* = 0 s, the TRM was in the cooler region, preparing for motion. By *t* = 29 s, it had reached the hot detection zone and remained stationary to perform temperature sensing. After a period of stable sensing, at *t* = 269 s, the TRM completed the detection process and began moving to the next area. Finally, at *t* = 332 s, the TRM navigated obstacles and successfully arrived at the cooler recovery zone. Notably, due to the irreversible nature of the TRM’s temperature sensing mechanism, the information gathered during sensing remained intact even after returning to the cooler zone (Movie S3). After recycling, the TRM image was processed using the trained neural network model to determine the temperature of the high-temperature detection zone. The predicted temperature was 198.73 °C, demonstrating the TRM’s effectiveness in accurately sensing high-temperature conditions.

We further evaluated the stability of the TRM system by conducting experiments under diverse operational conditions. The microrobots were tested in different preset temperatures (160 °C to 240 °C) using the same confined-space simulator. At each temperature, the TRMs were independently tested and analyzed. Representative results are shown in Fig. 5C, with the batch B1. In addition, inter-batch validation was carried out to assess the reproducibility of the system. Five independently prepared TRM batches were tested under identical conditions, and their measured temperature values exhibited strong consistency, as shown in Fig. 5C (batches B2 to B6). These results confirm the reliability of the neural network model and the reproducibility of the TRM fabrication process. Together, these validation experiments highlight the TRM’s potential for accurate and stable temperature sensing in confined and extreme environments.

Additionally, to explore the feasibility of TRM deployment in nontransparent and confined environments, we first demonstrated its maneuverability under limited-visibility conditions (Figs. S9 and S10 and Movies S4 and S5). Subsequently, we performed a temperature sensing experiment using a representative model of a confined, nontransparent environment—a porous silicon carbide structure. As shown in Fig. 5D, the bottom of the porous structure was heated to 200 °C. The TRMs were initially positioned on the surface and navigated laterally across the structure. Guided by a directional magnetic field, they were then steered downward through the internal pores to reach the heated bottom region for temperature sensing. After sensing, the TRMs continued to move along the same magnetic trajectory and exited from the bottom opening for recovery. Figure 5E illustrates the time-resolved sequence of this process. At *t* = 0 s, the TRMs were located on the surface and began leftward movement toward a target pore, which, as noted in Fig. S11, was not a through-hole. Based on a simple evaluation of the internal layout, the magnetic field was redirected downward at *t* = 10 s, guiding the TRMs through the structure. After they reached the designated sensing region and completed the sensing task, the TRMs continued along the same trajectory and exited the bottom of the structure at *t* = 276 s, enabling post-task recovery. The recovered TRMs were analyzed collectively by averaging their colorimetric features and processing them through the trained MLP model, yielding a predicted temperature of 199.76 °C. This result confirms the TRM’s effective sensing performance.

## Conclusion

In summary, this study offers a distinct approach to high-temperature sensing in spatially constrained and optically inaccessible environments by using microrobot. The microrobot demonstrates a sensing range of 160 to 240 °C, with its color transitioning from blue–white at 160 °C to deep yellow at 240 °C. Its combination of magnetic actuation, passive operation, irreversible high-temperature sensing, and neural network analysis enables a sensing mode for confined, high-temperature environments.

This study investigated the mechanism underlying the microrobot’s temperature sensitivity and successfully fabricated Janus microrobots using microfluidic technology. Systematic experimental validation and characterization confirmed the temperature-sensing performance of the microrobots at different temperatures. Furthermore, an efficient temperature-sensing strategy was developed using the MLP neural network model, enabling fast and convenient temperature measurement. The findings of this research provide a novel method for using microrobots to measure temperature in the core regions of complex constrained environments. The experimental demonstrations in porous microchannel environments simulate internal temperature detection within geological-like microstructures. These tests validate the feasibility of using microrobots to perform thermal sensing in confined, nontransparent domains, particularly under low or nonmagnetic background conditions. Future work will explore expanding the sensing range, miniaturizing TRM size, improving multi-zone detection through sensor swarms, and optimizing the Helmholtz coil system to enhance magnetic field uniformity and penetration depth.

## Materials and Methods

### Material sources and characteristics

Copper(II) sulfate pentahydrate, polyethylene–polypropylene glycol (PluronicF127) ammonia, and anhydrous ethanol were all purchased from Shanghai Aladdin Biochemical Technology Co., Ltd.; high-temperature-resistant photocurable resin was obtained from Hubei Jinchao Software Technology Co., Ltd.; glycerol was purchased from Guangzhou Xilong Chemical Co., Ltd.

### Preparation of dispersed phase solutions

Copper(II) sulfate pentahydrate crystals were ground in a ball mill for 18 h to obtain copper sulfate powder with a particle size of less than 1 μm. First, the ground copper(II) sulfate pentahydrate particles were dissolved in 10 ml of deionized water, followed by the slow addition of 10 ml of ammonia solution, resulting in the formation of a blue precipitate. The mixture was stirred continuously to ensure complete reaction. Subsequently, 25 ml of absolute ethanol was added to facilitate the precipitation, and the precipitate was collected. To obtain high-purity tetraamminecopper(II) sulfate solid, the precipitate was alternately washed with ammonia solution and absolute ethanol. Next, 2 g of the tetraamminecopper(II) sulfate solid was finely ground and dispersed in 8 g of high-temperature-resistant resin. The mixture was initially stirred manually for 3 min to ensure preliminary mixing, followed by 10 min of ultrasonic treatment to enhance dispersion uniformity. Finally, the solution was mechanically stirred at 900 rpm for 90 min, yielding a stable and homogeneous dispersed-phase solution suitable for microfluidic applications.

### Preparation of continuous phase solutions

A total of 1 g of PluronicF127 was dissolved in 100 ml of purified water and ultrasonicated for 60 min to ensure complete dissolution. Subsequently, 50 g of glycerol was gradually added, followed by an additional 30 min of ultrasonic dispersion to promote homogeneous mixing. Finally, the resulting solution was filtered through a 0.45-μm aqueous-phase filter to remove impurities, yielding a high-purity continuous-phase solution.

### Experimental setups and systems

The magnetic field was generated using a custom-built 3D Helmholtz coil magnetic control system. This system provides a uniform alternating magnetic field in arbitrary directions, with a frequency range of 0 to 60 Hz and a magnetic field strength range of 0 to 30 mT. The effective uniform field region measures 5 cm × 7 cm × 3 cm. The magnetic control system is compatible with an Olympus upright stereo microscope (Phenix, XTL-165-VT) and is equipped with a charge-coupled device camera (FLIR-GS3-U3-51S5C-C) for capturing and recording object motion. A high-pressure syringe pump (Longer, LSP01-1BH) was used to precisely control the flow rate during microfluidic experiments. An ultra-depth digital microscope (Keyence, VHX-900F) was employed to observe the color changes of the microrobots. Image processing and data analysis were performed using ImageJ software (National Institutes of Health).

## Data Availability

The data supporting this article have been included as part of the ESI and are available from the corresponding authors.
