# Supplementary material for: Temperature-Responsive Microrobot for High-Temperature Sensing in Constrained Environments
Source: Research (Wash D C). 2025 Jul 4;8:0760. doi: 10.34133/research.0760 (PMC12231237; doi:10.34133/research.0760)
Supplement: Supplementary 1 — Figs. S1 to S11 Table S1 Movies S1 to S6 [file research.0760.f1.zip › Supporting Materials_Revised_Clean.pdf]

Supporting Information

Temperature Responsive Microrobot for High-Temperature Sensing in Constrained Environments

Shaobo Ding[1,2†], Junmin Liu[1,2†], Jiaxu Dong[1†], Rencheng Zhuang[1], Enbo Shi[1], Shutong Wang[1], Yuhang Xiao[1], Dekai Zhou[1,2*], Longqiu Li[1,2*] and Xiaocong Chang[1,3*]

1. Key Laboratory of Microsystems and Microstructures Manufacturing (Harbin Institute of Technology), Ministry of Education, Harbin 150001, China.

2. Zhengzhou Research Institute of Harbin Institute of Technology, Zhengzhou, 450000, China.

3. Chongqing Research Institute of Harbin Institute of Technology, Chongqing, 400722, China.

*Address correspondence to: changxiaoconghit@163.com

†The authors contributed equally to this work.

E-mail: dekaizhou@hit.edu.cn, longqiuli@hit.edu.cn, changxiaoconghit@163.com

List of Videos:

Movie S1. Trajectory of the TRM.

Movie S2. Motion of TRMs.

Movie S3. The whole detection process of the TRM.

Movie S4. Oriented magnetic field direction motion of the TRMs in a nonvisual environment.

Movie S5. Oriented magnetic field direction motion of the TRMs in a visual environment.

Movie S6. TRMs perform temperature measurement in non-transparent and porous structures.

Table. S1 Comparison of different temperature measurement methods.

Sensing Method	Temp. Range (°C)	Confined Access	Detection Mode	Reversibility	Resolution	Response Time	Flexibility Level	Cost Level
Thermocouple	-270 to 1800	Contact only	Wired data acquisition	-	±0.1~ ±1 °C	0.1~10s	Low	Low–Medium
Infrared Thermometry	-50 to 1000	LOS needed	Real-time IR camera	-	±0.1~ ±1 °C	240 ms – µs	Medium	Medium–High
Optical Fiber	-800 to 600	Limited	Real-time optical readout	-	0.01~ 0.1 °C	µs–ms	Medium–High	Medium–High
Quantum Dots	-100 to 200	Subcellular	Fluorescence emission (in situ)	Yes	0.1~1 K	ms	Medium–High	Medium–High
Lanthanide-doped NPs	-30 to 500	Tissue scale	Upconversion/ fluorescence	Yes	mK	µs	Medium–High	High
NV Centers in Diamond	-190 to 300	Intracellular	ODMR with laser & RF	Yes	mK	s	Low	Very High
HT-NRs	38 to 45	100 µm	Photothermochromism	Yes	1 °C	1s	High	Medium–High
TACSI microrobot	37 to 60	50µm	thermoresponsive fluorescent dye	Yes	2 °C	0.1s	High	Medium–High
TRM	160 to 240	100 µm	offline sensing and retrieval-based	No	±5°C	240s	High	Low–Medium

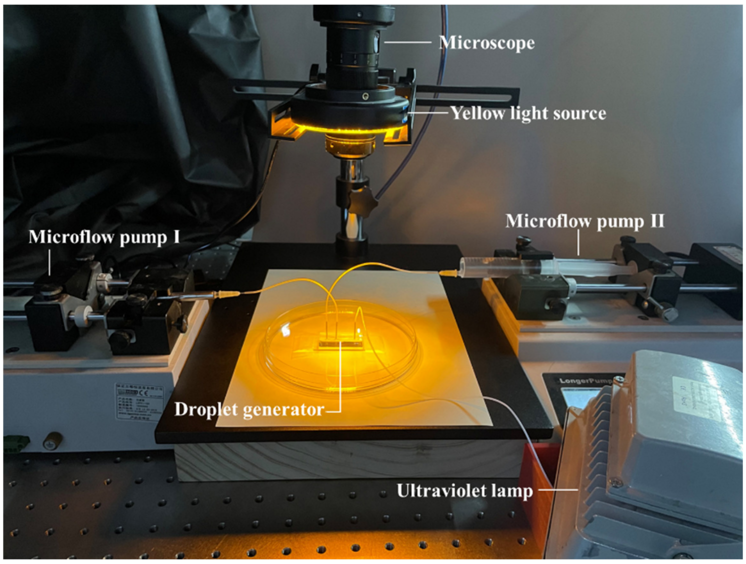

Fig. S1 Microfluidics-based microsphere generation system.

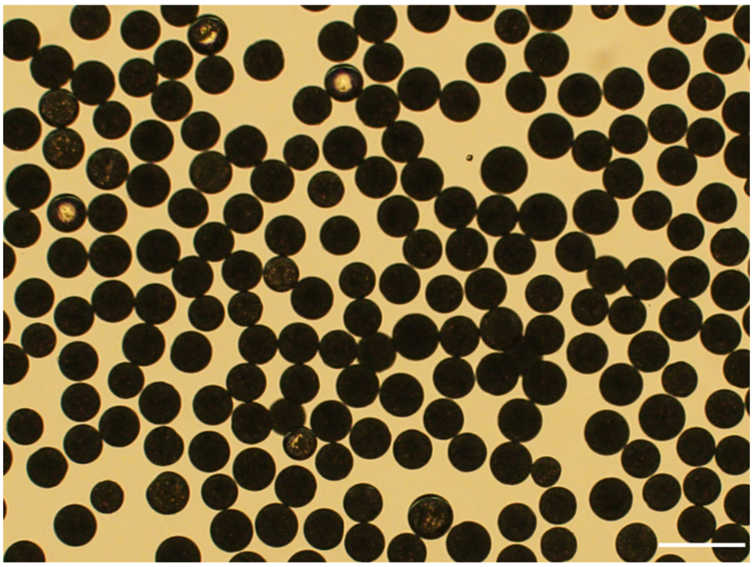

Fig. S2 Base microsphere under the microscope, scale bar represents 200 μm

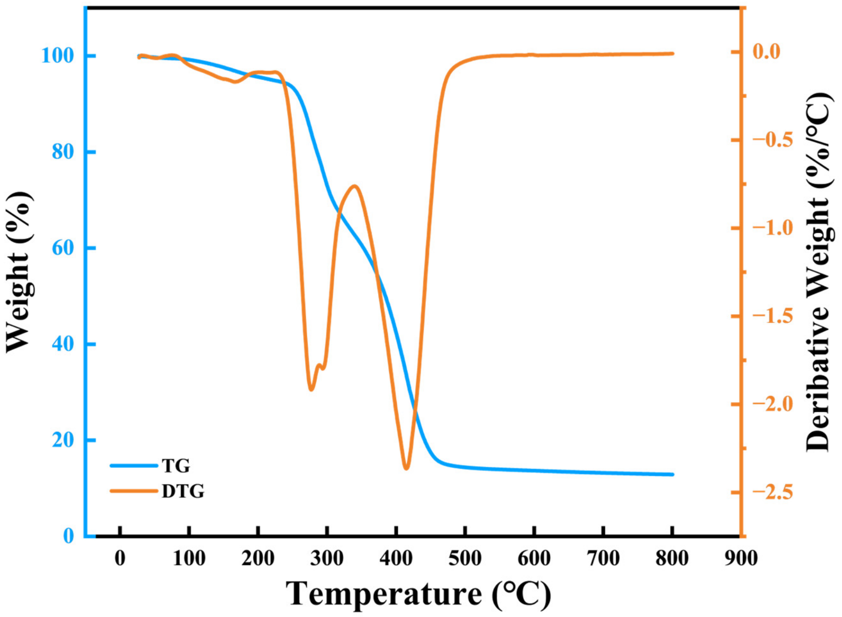

Fig. S3 Thermogravimetric (TG) curve of the base microsphere

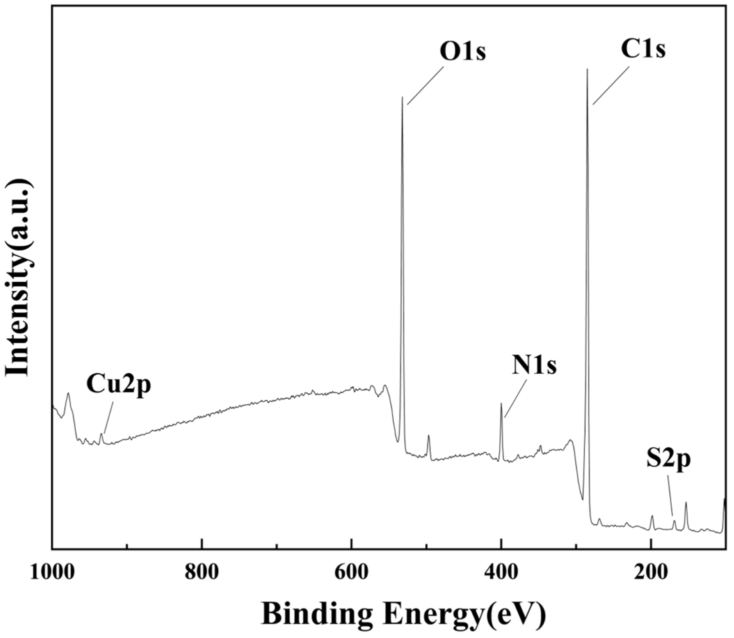

Fig. S4 X-ray Photoelectron Spectroscopy (XPS) analysis of the base microsphere

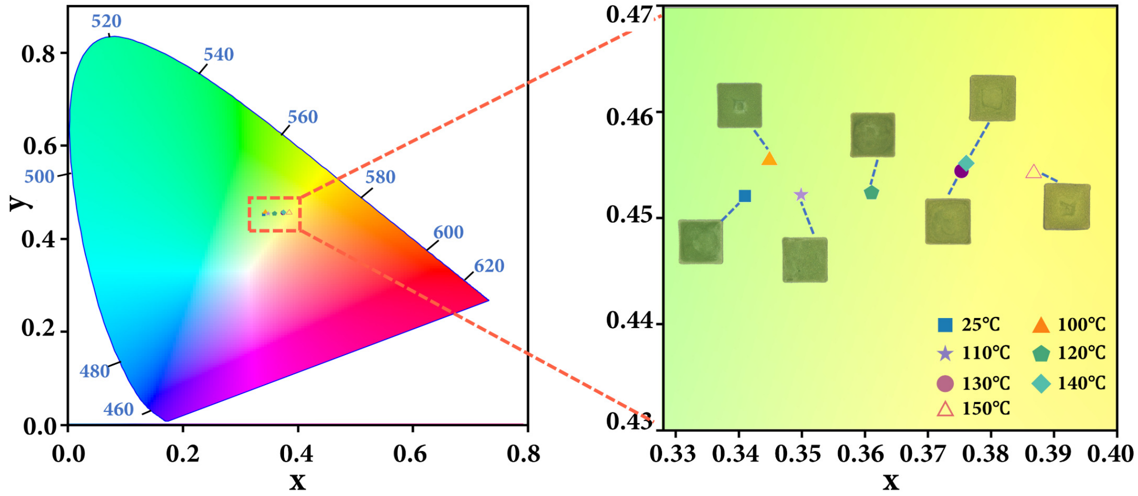

Fig. S5 A simplified example of thermochromism: loss of water of crystallization in nickel(II) chloride hexahydrate leading to colour change.

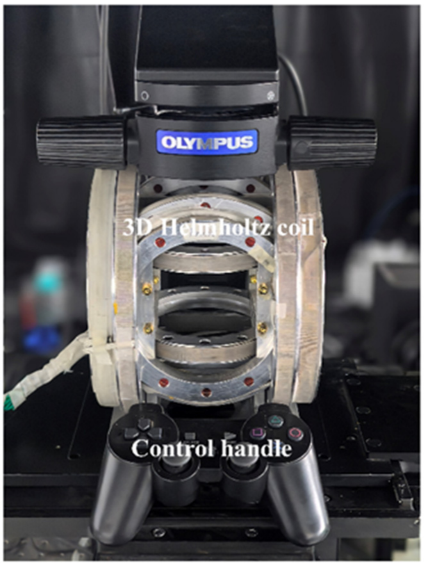

Fig. S6 3D Helmholtz coil magnetron system.

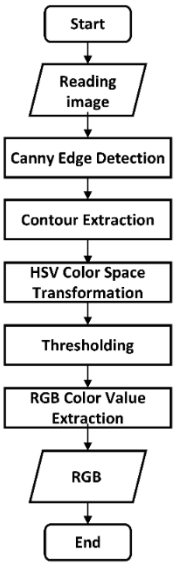

Fig. S7 Color extraction flow chart.

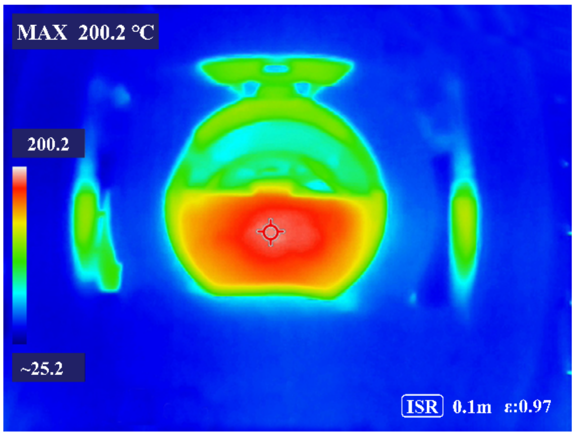

Fig. S8 Infrared temperature measurement experiment image.

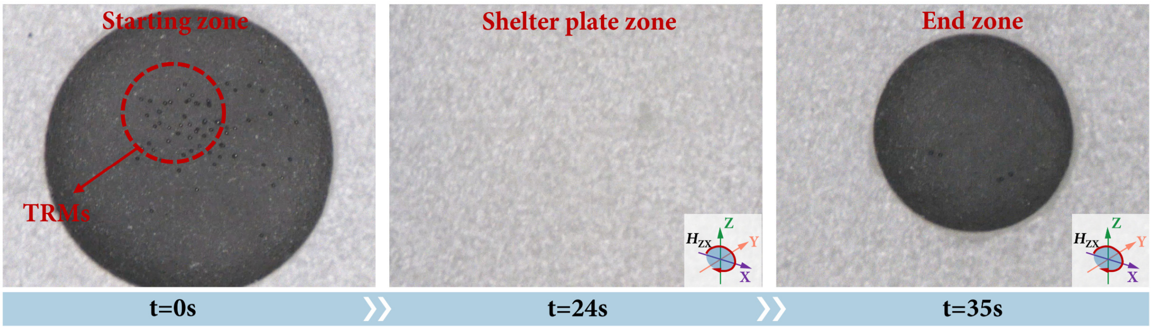

Fig. S9 Motion of the TRMs with an opaque mask under a directional magnetic field.

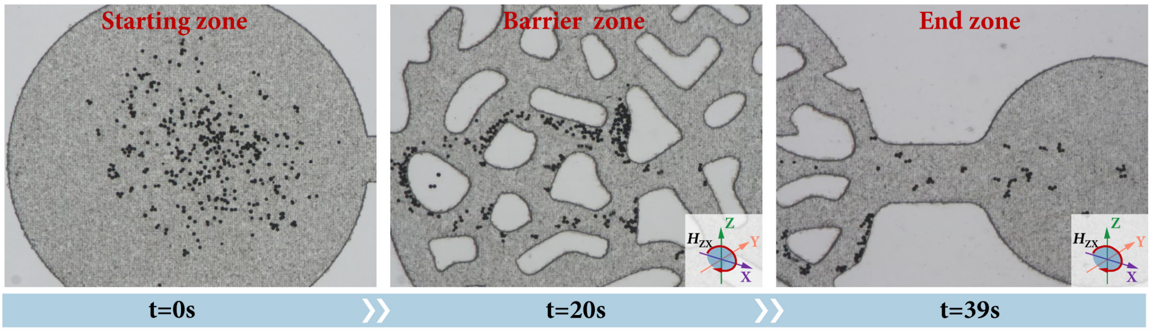

Fig. S10 Motion of the TRMs without an opaque mask under a directional magnetic field.

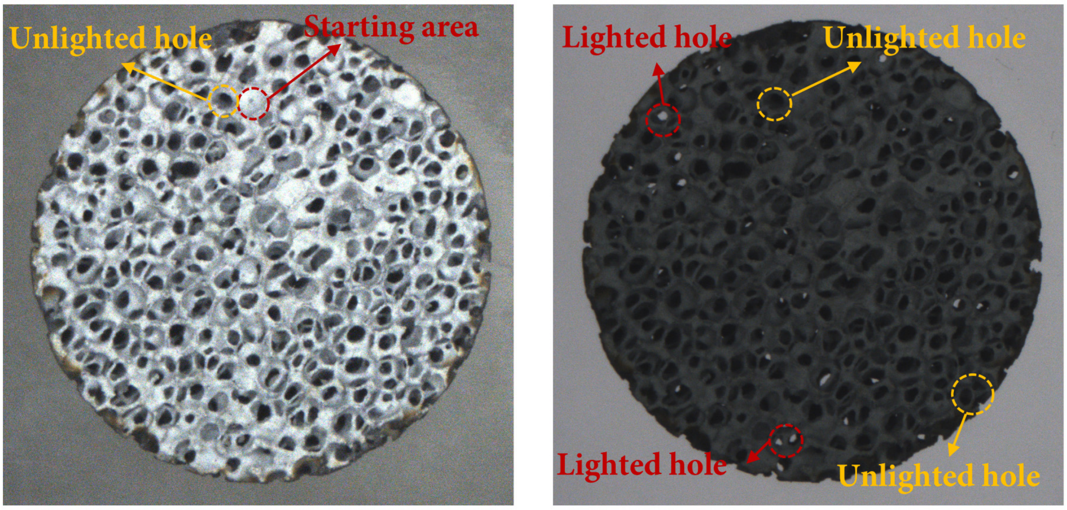

Fig. S11 Description of target hole structure.
